# Emerging role of serum biomarkers in neurodegenerative dementias in a diverse context

**DOI:** 10.1002/alz.090260

**Published:** 2025-01-09

**Authors:** Faheem Arshad, Sarada Subramanian, Subasree Ramakrishnan, Suvarna Alladi

**Affiliations:** ^1^ National Institute of Mental Health and Neurosciences [NIMHANS], Bengaluru, Karnataka India; ^2^ National Institute of Mental Health and Neurosciences (NIMHANS), Bengaluru India; ^3^ National Institute of Mental Health and Neurosciences, Bangalore India

## Abstract

**Background:**

The aetiology of Alzheimer’s Disease and Related dementias (ADRD) in a diverse context is complex, and clinical methods lack sensitivity and specificity due to overlapping symptoms. This underscores the need of novel serum biomarkers in diagnosis of dementia. Serum glial fibrillary acidic protein (GFAP) and neurofilament light chain (NfL) are non‐amyloid blood‐based biomarkers indicative of ongoing inflammatory and neurodegenerative process. We aimed to assess and validate their diagnostic value in patients with Alzheimer’s dementia (AD) and Frontotemporal dementia (FTD) spectrum disorders in the Indian context.

**Method:**

Ninety‐nine participants recruited in the study underwent detailed neurocognitive assessment along with basic brain imaging. Quantitative determination of serum Glial fibrillary acidic protein (GFAP), neurofilament light chain (NfL), total‐tau and ubiquitin C‐terminal hydrolase L1 (UCHL1) were assessed using Simoa platform following a 2‐step digital immunoassay method as per manufacturer’s instructions using HD‐X analyser. Healthy controls (n=29), and participants with AD (n=28) and FTD spectrum disorders (n=42) diagnosed as per standard criteria were recruited.

**Result:**

Mean age of the participants and healthy controls was 61.26 years (10.31) and 55.69 years (8.97), respectively. GFAP and NfL were significantly higher in participants with dementia compared to healthy controls (p<0.001). Serum GFAP levels were higher in AD (p<0.001), while NfL levels were significantly elevated in AD and FTD compared to controls (p< 0.05). However, there was no difference in t‐tau and UCHL1 levels between dementia group and healthy controls. Higher biomarkers levels of serum GFAP, NfL, t‐tau and UCHL1 were significantly associated with increasing levels of severity of dementia (r=0.539, p=0.000; r=0.637, p=0.000; r=0.241, p=0.016; r=0.476, p=0.000; respectively).

**Conclusion:**

Levels of GFAP and NfL in serum have an emerging role in differentiating ADRD (AD and FTD) from healthy controls. These biomarkers are valuable predictors of severity across the neurodegenerative dementias. Further prospective cohort studies are required to replicate these findings in a diverse context.

